# Hair Dye

**Published:** 1884-10

**Authors:** 


### A NEWLY-DISCOVERED HAIR DYE.

THE "Moniteur Scientifique" describes a new dye, said to have a progressive action, to produce all shades up to deep chestnut color, and yet to be free from all deleterious action. The base of the dye is bismuth. Following is the formula—Bismuth is dissolved in the smallest possible quantity of nitric acid, nearly three parts, and to this liquid a solution in water of tartaric acid, equal in weight to one-fourth of the bismuth used, is added, and then a large quantity of water. The precipitate is filtered off and washed with water until the washings have lost all acidity. The precipitate is dissolved in a solution of ammonia; more than a fluid ounce of solution of ammonia will be required for each oz. of bismuth. Hyposulphite of soda, three-fourths of the weight of solutiou used, is then added, and when the salt is dissolved the mixture is filtered and preserved in well-closed bottles. The dye should contain about one-twentieth of its weight of bismuth. Such a mixture is said to form an admirable dye.

